# Regulation of adipocyte autophagy — The potential anti-obesity mechanism of high density lipoprotein and ApolipoproteinA-I

**DOI:** 10.1186/1476-511X-11-131

**Published:** 2012-10-06

**Authors:** Shuai Wang, Daoquan Peng

**Affiliations:** 1 Department of Cardiology, The Second Xiangya Hospital of Central South University, 139 Middle Ren-Min Rd., Changsha, Hunan, 410011, China

**Keywords:** HDL/apoA-I, Obesity, Autophagy, Adipocyte

## Abstract

Obesity is reaching epidemic worldwide and is risk factor for cardiovascular disease and type 2 diabetes. Although plasma high density lipoprotein (HDL) and apolipoprotein A-I (apoA-I) are inversely correlated to obesity, whether HDLs have anti-obesity effect remains unclear until a recent study reporting the direct anti-obesity effect of apoA-I and its mimetic peptide. However, the mechanism is not fully understood. Increasing adipose energy expenditure through attainment of brown adipocyte phenotype in white adipose tissue is considered a potential strategy to combat obesity. Specific inhibition of autophagy in adipose tissue is associated with reduced adiposity which is attributed to the attainment of brown adipocyte phenotype in white adipose tissue and the increased energy expenditure. HDL and apoA-I could activate PI3K-Akt-mTORC1 signaling which negatively regulates autophagy. The links between HDL/apoA-I and autophagy brings a new understanding on the anti-obesity effect of HDL and apoA-I.

## Introduction

Obesity is the most common nutritional disorder and is associated with important comorbidities such as dyslipidemia, atherosclerosis, type 2 diabetes and insulin resistance [1]. Anti-obesity pharmacotherapy is a potentially important adjunctive treatment to lifestyle modification. Drugs used to induce weigh loss may act through reducing appetite and increase satiety (e.g. sibutramine and rimonabant), reduce the absorption of nutrients (e.g. orlistat) [2]. Amongst the drugs marketed for weight loss there have been many instances of market withdrawl due to adverse events, leaving only orlistat approved for long term use [3]. The selective cannabinoid 1-receptor blocker Rimonabant, once considered as a promising anti-obesity drug which could improve dyslipidemia associated with the metabolic syndrome, including raising HDL and reducing TG, has been withdrawn recently from the market because of psychiatric adverse events [3-5]. Another problem with anti-obesity drugs is lack of data on major obesity-related morbidity and mortality. Therefore, development of effective and safe drugs is an area of intense clinical interest.

The well-established cardioprotective nature of high-density lipoproteins (HDLs) has made them and their main protein apolipoprotein A-I (apoA-I) popular targets for potential cardiovascular therapies. Current HDL-based therapies including direct infusion of rHDL and apoA-I mimetic peptides have exhibted abroad beneficial effects aside from mediating cholesterol efflux such as anti-inflammatory, anti-oxidative actions and shown potential usefulness as therapy for diseases involving chronic inflammation and oxidative stress [6].

One of the proatherogenic effects of obesity is attributable to its accompanying dyslipidemia. The prominent dyslipidemia in obesity is low high density lipoprotein cholesterol (HDL-C) levels and apoA-I. Epidemiological studies have shown a strong inverse correlation between HDL-C, apoA-I and obesity, especially in individuals with visceral obesity [7]. This inverse correlation was originally attributed to the disturbed metabolism of HDL and apoA-I in obesity status. But recent studies suggested that HDL/ApoA-I had reciprocal effect on obesity. This article will focus on the updated understanding of the anti-obesity effect of HDL and apoA-I.

### Anti-obesity effect of apoA-I

Obesity is defined medically as a state of increased body weight, more specifically adipose tissue, or sufficient magnitude to produce adverse health consequence [8]. As the body’s largest energy reservoir, adipose tissue serves the primary function of lipid storage in the fed state. In fasting state, fatty acid is released from the breakdown of triglyceride (TG) into circulation for energy production [9]. Prolonged energy imbalance between energy intake and expenditure leads to an increase in both fat cell size and fat cell number [10]. Large adipocytes especially adipocytes present in visceral fat have a higher rate of lipolysis. It is known that obesity clearly associates with increased circulating free fatty acids (FFAs) and adipocytokines which not only initiate adipose inflammation but also drive all aspects of metabolic syndrome, including insulin resistance, dyslipidemia, and hypertension, eventually leading to increased risk for cardiovascular diseases [1].

HDL is generally considered as a protective factor for cardiovascular disease. In addition to the well known effect of mediating reverse cholesterol transport, it also exerts other beneficial functions such as anti-oxidative, anti-inflammatory and anti-thrombotic actions [11]. It’s well known that obese individuals display lower plasma levels of HDL-cholesterol and apoA-I, with HDL-cholesterol levels associated with both degree and distribution of obesity [7,8,12].

Previous findings that several polymorphrism in apolipoprotein A1(ApoA-I) gene have been associated with obesity in Brazilian population and that body fat content was increased in apoA-I null mice model lead to query about the role apoA-I played in obesity development [13,14]. Several recent studies aimed to answer this question studied the role of apoA-I in regulating obesity through two main lines of investigation: (1)overexpression of genes that encode apoA-I in mouse models; (2) administration of apoA-I mimetics D-4F and L-4F, which share structural and biological features of native apolipoprotein A-I, to mimic increased plasma apoA-I level in mouse models. Diet-induced obesity (DIB) was generated in apoA-I transgenic (ApoA-I-Tg) and wild type mice by feeding high fat diet for three months. Although both groups of mice had the same body weight gain and food intake, the body fat content was significantly lower in ApoA-I-Tg mice than the wild-type mice as evidenced by reduced weight of epididymal and retroperitoneal fat pat. Consistent with this result, in other studies, daily administration of D-4F and L-4F in high fat diet fed mice reduced weight gain and decreased obesity associated hyperglycemia when compared with age-matched vehicle-treated obese mice [15,16]. Taken together, these observations implicated a potential anti-obesity effect of apoA-I.

Up to date, there are several strategies for pharmacotherapy of obesity, including: (1) appetite suppression through central stimulation of anorexigenic signals or blocking orexigenic signals; (2) inhibition of nutrient digestion and absorption through gastrointestinal mechanism; (3) stimulation of fat mobilization and decreasing triacylglycerol synthesis and deposition in fat depots; (4) increase lipid oxidation or thermogenesis by uncoupling fuel metabolism, thereby enhancing energy expenditure [17]. In the above mentioned studies, the anti-obesity effect of apoA-I was not associated with a reduction of food intake or increased locomotive movement. Investigation into the metabolic profile confirmed enhanced energy expenditure through increased respiratory exchange ratio with consistent increased expression of uncoupled protein 1 (UCP1) in brown adipose tissue [16]. UCP1 expresses specifically in brown adipose tissue and is responsible for detaching fatty acid oxidation from the coupling to respiratory chain, resulting in thermogenesis [18]. It has been demonstrated that increased expression of UCP1 in brown adipocyte or ectopic expression of UCP1 in mouse or human skeletal muscle and white adipocyte promotes fatty acid oxidation and resistance to obesity [19]. Intriguingly, ApoA-I gene overexpression and D-4F treatment lead to significantly increased expression of UCP1 in brown adipose tissue [16]. Therefore, UCP1 is probably one of the target genes regulated by apoA-I in brown fat, and such regulation might contribute to the increase of energy expenditure observed in apoA-I over-expression and mimetic peptide treated mice. However, the mechanism underlying the anti-obesity effect of apoA-I remains to be further elucidated.

## Autophagy and obesity

### Phenotype transition from WAT to BAT

Adipose tissue contains two distinct types of fat cells, white and brown. The balance between white and brown adipose tissue (BAT) is a factor that determines obesity. White fat cells are specialized for the storage of chemical energy as triglycerides, while brown fat cells have very limited ability to store lipid and serve the primary function of dissipating chemical energy through adaptive thermogenesis [20]. Since brown adipocyte is more oxidative, BAT is generally considered as an organ counteracting obesity. Generally, proneness to obesity and metabolic disease correlates with decreased BAT activity, whereas resistance to obesity correlates with increased BAT function or the induction of brown adipocyte-like gene expression in white adipose tissue [21-27]. However, BAT was previously considered to be important only in small mammals such as rodent because in human, BAT was previously thought to disappear soon after birth [20]. Not until recent findings of presence of substantial amounts of metabolically active brown adipose in healthy adult human that the importance of BAT in metabolism has been recognized and that the speculation as to whether controlled recruitment of brown adipocyte would be a potential strategy to combat obesity has been raised [21,28-31].

Ample evidences support some plasticity exists between white and brown adipocyte. In mice and rats, exposure to cold or β-adrenergic agonists induces the appearance of brown adipocyte in traditional white fat pats, which originate differently from the classic brown adipocyte that develop before birth because these newly formed brown adipocyte did not express the YFP reporter gene, which was conspicuously expressed in the interscapular brown fat cells from the same mice [32-35]. Similarly, in human, working in the cold temperature could induce occurrence of brown adipose tissue in outdoor workers [36]. Some pathological states such as pheochromocytoma, hibernoma, and diseases involving chronic hypoxia, Chagas’ disease, Duchenne dystrophy and cancers, are known to stimulate BAT proliferation in white pats [37]. These evidences support the possibility that under certain conditions,cell with the morphology and molecular phenotype of brown fat can be induced in WAT. In addition, given that BAT and WAT are both present in various adipose tissue depots in human, attainment of a brown adipocyte cell phenotype in white adipocyte may be a potential strategy for combating obesity.

### Autophagy regulate obesity through phenotype transition

Autophagy, defined as a highly regulated process involving the bulk degradation of cytoplasmic macromolecules and organelles in mammalian cells via the lysosomal system, plays a housekeeping role in removing misfolded or aggregated proteins, clearing damaged organelles, eliminating intracellular pathogens, and balancing sources of energy at critical times in development and in response to nutrient stress [38,39]. As a survival mechanism, dysregulated autophagy has been linked to many human pathophysiologies, such as cancer, myopathies, neurodegeneration, heart and liver diseases [40]. Interestingly, recent studies revealed that autophagy was upregulated in obese individuals, as evidenced by increased expression of autophagy gene Atg5, LC3A, and LC3B as well as elevated autophagic flux in omental and subcutaneous adipose tissue [41]. Consistent with this conclusion, other group reported that autophagy was strongly up-regulated in patient with diagnosis of Type 2 diabetes (T2D) and overweight [42]. This relationship between increased autophagy and obesity lead to a question that what is the role autophagy play in the state of obesity. On the one hand, excess lipid storage in hypertrophied adipocyte leads to increased endocytoplasmic reticulum (ER) activity, which ultimately overwhelms the capacity of ER to properly fold nascent protein, therefore causing ER stress and subsequent oxidative stress in the mitochondria, FFA release and proinflammatory state of adipoctye, which all impinge on mTOR and induce autophagy [43]. In this sense, activated autophagy may serve as a protective mechanism necessary for cell survival in the challenging environment that develops in adipose tissue. On the other hand, increased autophagy possibly signifies a process underlying cell death of hypertrophied adipocyte [44].

**Figure 1:**
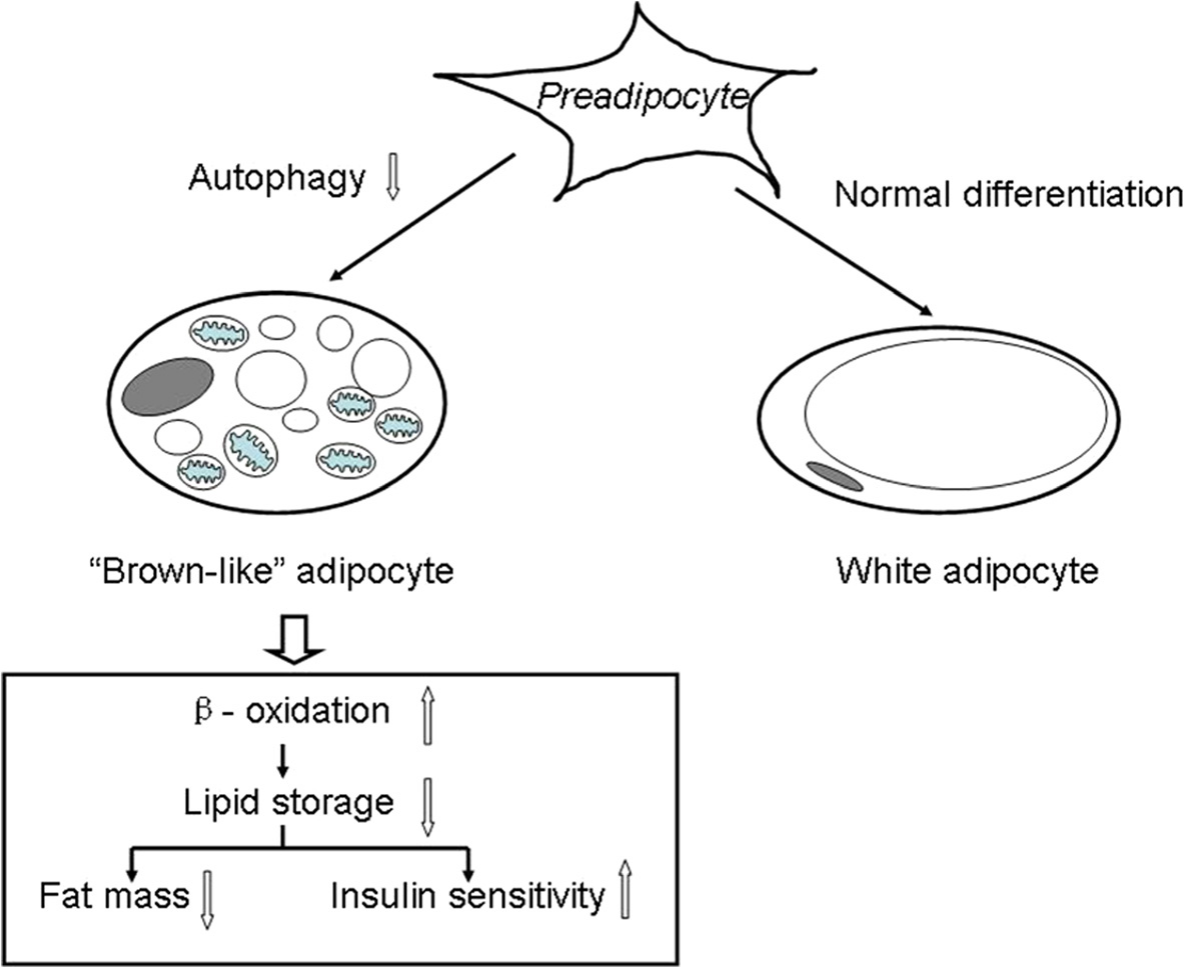
**Autophagy regulated obesity through phenotype change.** Loss of autophagy in preadipocyte altered the normal differentiation process to white adipocyte and resulted in “brown-like” adipocyte, which was smaller with multilocular lipid droplet and more mitochondria. Metabolically, it had increased energy consumption through free fatty acid β-oxidation with consequently reduced lipid storage, less fat mass and increased insulin sensitivity.

To further elucidate the role of autophagy in the state of obesity, recent studies used knockout mice model with adipose tissue specific deletion of autophagy-related gene Atg7 and Atg5 respectively. The Autophagy-related gene knockout mice exhibited a metabolic favorable phenotype. The mutant mice were slimmer with increased insulin sensitivity and showed resistance to high-fat-diet induced obesity compared with wide-type mice. The mutant mice contain only 20% of the mass of WAT found in wild-type mice [40,45,46]. Consistent with this finding, in vitro studies using cell line preadipoctye 3T3-L1 and primary MEFs showed that autophagy inhibition through Atg7 knockout or 3-MA treatment blocked adipocyte differentiation [9]. Thus, it seems that the metabolic favorable phenotype generated in Atg7/Atg5 knockout mice may result from a blocking in white adipocyte differentiaton which leads to a failure of these cells to accumulate lipid. However, the decreased serum free fatty acids and the absence of accumulation of excess lipid in nonadipose organs such as liver and heart in mutant mice did not support this proposition. Because WAT served as body’s main reservoir of lipids, if the WAT capacity of lipid storage was simply blocked without any increased energy expenditure, detrimental elevation of serum FFA and ectopic lipid deposition would occur. Actually, what’s interesting in those studies was that most of the mutant white adipocyte showed some characteristics of brown adipocytes. They were smaller, multilocular and contain more mitochondria. These cells also exhibited altered fatty acid metabolism with increased rates of β-oxidation and reduced rates of hormone-induced lipolysis. In addition, brown adipocyte specifically expressed UCP1 was found in WAT of Atg7-knockout mice [45]. Former studies have demonstrated that exogenous overexpression of PGC-1α, a critical mediator of brown fat differentiation, induced the development of features of brown fat in WAT [47]. In autophagy-related gene-knockout mice, PGC-1αwas found to be induced in WAT [9]. Loss of autophagy induced brown-like adipocyte in WAT may either through promoting progenitor cell shutting into the pathway of brown adipocyte differentiation or altering adipocyte transdifferentiation by promoting the conversion of WAT into BAT or blocking the reverse process. Since recent evidence support that WAT and BAT have different precursors and that in the knockout studies, younger mice (3 weeks) failed to show the phenotypic change that was observed in older mice (12 weeks), it is most likely that the phenotypic changes occurred after original WAT formation and was attributed to a phenotype transition from “white to brown” [48]. As regard to how loss of autophagy mediated this phenotypic transition process, it has been known that in rodent, BAT and WAT expressed pre- and post-natal respectively [49]. In human, recent studies demonstrated a transient expression of UCP1 during adipogenesis of adipocyte derived stem cells (ADSCs), which indicate the possibility that ADSC pass through a brown adipocyte-like stage while differentiating into adipocyte [50]. Therefore, autophagy may be critical for removal and degradation of excessive mitochondria or proteins which represent features of brown adipocyte during adiopogenesis. Downregulated autophagy in preadipocyte altered the normal process of adipocyte differentiation and resulted in adipocyte resembling brown adipocyte which has increased ability of energy consumption (Figure 1). 

### Regulation of autophagy

One of the major roles of autophagy is to serve as a cellular adaptive reaction in order to sustain the internal organization in the case of insufficient external nutrient supply or augmented energy demands through digesting their own interior. On the one hand, autophagy needs to be upregulated under nutrient restriction. On the other hand, cells have to avoid excessive and enduring self-digestion. In order to keep the delicate balance between external energy and nutrient supply and internal consumption, complex regulatory network that sense the environmental change is required.

The nutrient sensing kinase mTORC1 is well known to negatively regulate the autophagic machinery. Diverse external signals such as growth factor, amino acids, normoxia, or high energy levels all activate mTORC1 and result in the inhibition of autophagy [51-53]. mTORC1 inhibition, as resulting from rapamycin treatment was surposed to resulted in induction of autophagy. In mammalian cells, however, rapamycin treatment failed to induce autophagy as observed in yeast. This discrepancy is attributed to the rapamycin-resistant function of mTORC1. Although rapamycin fully inhibits mTORC1- dependent phosphorylation of S6K1, it only partially inhibits phosphorylation of other known mTORC1 substrate [54]. As a consequence, some mTORC1 dependent function such as autophagy is left unaffected by rapamycin. Instead, complete inhibition of mTORC1 function by another ATP-competitive inhibitor, Torin-1, could induce autophagy in mammalian cells [55]. In the obesity status, whether the activity of mTORC1 is augmented or attenuated is in debate. Previous studies using high fat induce obesity mice model demonstrated increased phosphorylation of mTORC1 substrate S6K1 in adipose tissue, which might indicate hyperactivity of mTORC1 [56]. However, as mentioned above, S6K1 phosphorylation does not represent the full function of mTORC1 activity. In addition, other study showed attenuated mTORC1 signaling along with affected downstream effects of mTORC1 signaling such as upregulated autophagy and impaired mitochondria in human adipocyte from obese patient with Type 2 Diabetes [42]. The reason for these discrepant results is still not clear. It is possible that the degree of obesity or whether insulin resistance exists may account for it.

AMP-activated protein kinase (AMPK) is regarded as the main energy-sensing enzyme that promotes all varieties of catabolic pathways and blocks several anabolic pathways [57]. AMPK has been well known to be linked to regulation of autophagy. Under energy-lower conditions, AMPK is activated, leading to autophagy induction [58]. Activated AMPK was thought to induce autophagy both directly through its action on autophagy initiator UIK1 (unc-51-like kinase 1) and indirectly through its action on mTORC1. In case of alarming energy states, AMPK could counteract the mTORC1-mediated repression on autophagy by at least two mechanisms through its action on TSC2 or Raptor [52,59]. In the state of obesity where energy intake exceeds energy consumption, AMPK activity in adipose tissue is decreased. However, whether this change in AMPK signaling alone under the situation of obesity is sufficient to affect autophagy rate is still not clear.

Sterol depletion, a selective form of nutrient depletion, has been reported to induce autophagy. Recently, it was suggested that in the situation of sterol limitation, apart from the de novo synthesis and receptor-mediated uptake pathway, cell could recycle cytoplasmic lipid droplet as a source of cholesterol through the process of autophagy [60]. Sterol regulatory element-binding protein 2(SREBP2), which preferentially controls expression of many cholesterogenic genes, is attributable to the sensing of cholesterol requirement and act as a feedback mechanism to maintain sterol homeostasis [61]. Recent study showed that SREBP2 directly activated autophagy genes during sterol depletion. Besides, SREBP-2 knockdown in the condition of cholesterol depletion decreased autophagosome formation and lipid droplet association of autophagosome related protein LC3 [62]. Numerous studies have proposed that the dilution in membrane cholesterol in hypertrophied adipocye may be sensed as true cholesterol depletion. SREBP2 that is sensitive to membrane cholesterol depletion is selectively activated in hypertrophied adipocyte in several obesity mice models [63]. Although SREBP2 may not be a general regulator of autophagy taken that many other situations that trigger autophagy are independent of cell lipid metabolism and that changes in gene expression is not essential for the rapid initiation of autophagy, it is reasonable to presume that genes involved in the induction of autophagy be activated in response to conditions where cell cholesterol is limited through the regulation of SREBP2.

## HDL and autophagy

### HDL prevent autophagy

Taken that HDL and apoA-I are inversely correlated to obesity and that apoA-I exhibits a direct anti-obesity action, it is of great interest to answer the question whether HDL/apoA-I could modulate adipocyte autophagy. Under conditions of excessive nutrition such as obesity, ER becomes stressed and results in activation of the copying system, which is termed unfolding protein response (UPR), in many metabolic tissues such as adipose tissue. Autophagy is activated by UPR so that unfolded and misfolded proteins could be degraded [64]. Recent studies reported that HDLs prevent ER stress in human endothelial cells and pancreatic β cells by reducing ER signaling and restoring protein folding and trafficking [65-67]. Thus, it is reasonable to hypothesize that HDLs may affect autophagy rate through alleviation of ER stress under the situation when stimuli exists. Interestingly, there is direct evidence showing that HDLs are able to prevent autophagy triggered by ox-LDL in endothelium [67]. Additionally, a large body of evidences support that HDL could activate PI3K-Akt signaling [68-71], a well identified upstream signaling of mTORC1 activation, which negatively regulate autophagy.

### HDL activated PI3K signaling

A major upstream signaling of mTORC1 is the phosphatidylinositol 3-kinase (PI3K) pathway. The binding of growth factor or insulin to cell surface receptors activates PI3K, which converts the plasma membrane lipid PIP2 to PIP3, subsequently recruits PDK1 and Akt to the plasma. Following being phosphorylated by PDK1, activated Akt positively regulates mTORC1 through phosphorylation-dependent inhibition of TSC2 with consequent autophagy inhibition [72].

**Figure 2:**
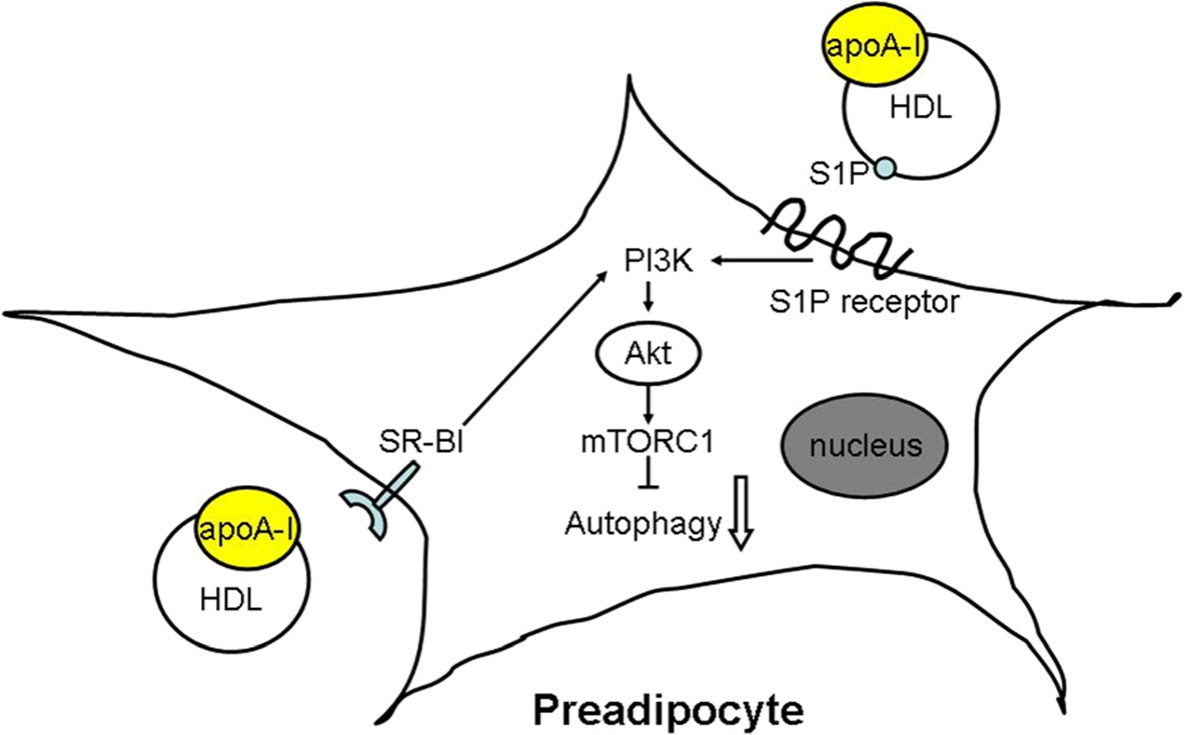
**Effect of HDL/apoA-I on autophagy in preadipocyte.** HDL/apoA-I activated PI3K-Akt signaling pathway through SR-BI dependent mechanism and/or S1P of HDL binding to S1P receptor. Activated Akt positively regulated the activity of mTORC1 with consequent autophagy inhibition. apoA-I, apolipoprotein A-I; HDL, high density lipoprotein; PI3K, phosphatidylinositol 3-kinase; S1P, sphingosine-1-phosphate.

Although numerous evidences showed HDL/apoA-I actived PI3K-Akt pathway in different cell lines, the underlying molecule mechanism still remains unclear. Rising evidences suggested that SR-BI, which mediated multiple antiatherogenic functions of HDL, was involved in PI3K-Akt signaling activation in response to HDL stimuli. Knock-down of SR-B1 by siRNA significantly attenuated HDL induced PI3K-Akt-eNOS signaling and prostacyclin production in endothelial cells [73]. Consistently, HDL stimulated glucose uptake in 3T3-L1 adipocytes involving PI3K-Akt activation via SR-B1. Knocking-down SR-B1 with RNA interference lead to diminish of glucose uptake stimulated by HDL with consistent pronounced inhibition of Akt activation [74]. Apart from SR-B1, sphingosine-1-phosphate (S1P) receptors binding with S1P of HDL has also been demonstrated to mediate PI3K-Akt signaling in mice myocardiocyte. A recent study demonstrated that HDL applied directly to isolated adult mouse cardiomyocytes enhances cell survival during hypoxia-reoxygenaration through stimulating signal PI3K-Akt. The prosurvival signal is mediated by S1P3 (sphingosine 1-phosphate 3) receptors located on the myocyte and are markedly attenuated by inhibitors of these receptors [71]. Taken together, HDL may activate PI3K-Akt signaling through SR-BI and S1P receptor (Figure 2). 

## Conclusion

Obesity, especially central obesity, is risk factor of cardiovascular disease and type 2 diabetes mellitus. Attainment of phenotype resembling brown adipocyte in WAT could reduce adipose deposit through enhanced β-oxidation. Inhibiting autophagy in adipose tissue leads white adipocyte to acquire features of brown adipocyte, therefore exerting an anti-obesity effect and improving obesity associated insulin sensitivity. Taken that HDL and apoA-I has direct anti-obesity effect in rat and that HDL activate PI3K-Akt signaling which negatively regulate autophagy through mTORC1, we propose that HDL/apoA-I inhibit autophagy through PI3K-Akt signaling, leading to phenotype transition of white adipocyte. This new link between HDL/apoA-I and autophagy provide a new understanding of the possible mechanism underline the antiobesity effect of HDL/apoA-I.

## Abbreviations

AMPK: AMP-activated protein kinase; apoA-I: Apolipoprotein A-I; apoA-I-Tg: apoA-I transgenic; BAT: Brown adipose tissue; C/EBP: CCAAT-enhancer-binding proteins; FFAs: Free fatty acid; HDL: High-density lipoprotein; LC3: Microtuble-associated protein light chain 3; mTORC1: Mammalian target of rapamycin complex 1; MEFs: Mouse embryonic fibroblast; oxLDLs: Oxidised low density lipoproteins; PDK: Phosphoinositide-dependent protein kinase; PI3K: Phosphoinositide 3-kinase; PPAR-γ: Peroxisome proliferator-activated receptor gamma; S1P: Sphingosine 1-phosphate; SR-B1: Scavenger receptor class B type I; SREBP: Sterol regulatory element-binding protein; TG: Triglyceride; TSC: Tuberous sclerosis complex; UCP: Uncoupling protein; UIK1 (unc-51-like kinase) WAT: White adipose tissue.

## Competing interests

The authors declare that they have no competing interests.

## Authors’ contributions

SW and DQP conceived the study, its design and drafted the manuscript. All authors read and approved the final manuscript.
